# Energy-Efficient PPG-Based Respiratory Rate Estimation Using Spiking Neural Networks

**DOI:** 10.3390/s24123980

**Published:** 2024-06-19

**Authors:** Geunbo Yang, Youngshin Kang, Peter H. Charlton, Panayiotis A. Kyriacou, Ko Keun Kim, Ling Li, Cheolsoo Park

**Affiliations:** 1 Department of Computer Engineering, Kwangwoon University, Seoul 01897, Republic of Korea; rmsqhwkd2@gmail.com (G.Y.); ysin0414@gmail.com (Y.K.); 2 Department of Public Health and Primary Care, University of Cambridge, Cambridge CB1 8RN, UK; pc657@medschl.cam.ac.uk; 3 Department of Engineering, School of Science and Technology (SST), City University of London, London EC1V 0HB, UK; p.kyriacou@city.ac.uk; 4 AI Lab, LG Electronics, Seoul 06763, Republic of Korea; kokeun.kim@lge.com

**Keywords:** spiking neural network, physiological signal, healthcare, photoplethysmogram, respiratory rate

## Abstract

Respiratory rate (RR) is a vital indicator for assessing the bodily functions and health status of patients. RR is a prominent parameter in the field of biomedical signal processing and is strongly associated with other vital signs such as blood pressure, heart rate, and heart rate variability. Various physiological signals, such as photoplethysmogram (PPG) signals, are used to extract respiratory information. RR is also estimated by detecting peak patterns and cycles in the signals through signal processing and deep-learning approaches. In this study, we propose an end-to-end RR estimation approach based on a third-generation artificial neural network model—spiking neural network. The proposed model employs PPG segments as inputs, and directly converts them into sequential spike events. This design aims to reduce information loss during the conversion of the input data into spike events. In addition, we use feedback-based integrate-and-fire neurons as the activation functions, which effectively transmit temporal information. The network is evaluated using the BIDMC respiratory dataset with three different window sizes (16, 32, and 64 s). The proposed model achieves mean absolute errors of 1.37 ± 0.04, 1.23 ± 0.03, and 1.15 ± 0.07 for the 16, 32, and 64 s window sizes, respectively. Furthermore, it demonstrates superior energy efficiency compared with other deep learning models. This study demonstrates the potential of the spiking neural networks for RR monitoring, offering a novel approach for RR estimation from the PPG signal.

## 1. Introduction

Respiration is a fundamental biological process that absorbs oxygen and eliminates carbon dioxide via the process of inhalation and exhalation [1]. Respiratory information provides reliable data for detecting changes in a patient’s health status, along with other vital signs such as body temperature (BT), blood pressure (BP), and heart rate (HR). In particular, respiratory rate (RR), which is the number of breaths per minute, is an important indicator obtained from various biomedical signals, such as photoplethysmogram (PPG) and electrocardiogram (ECG) signals, to assess the clinical status of patients. In addition, the variability of the continuous respiratory signal could be utilized to prevent not only respiratory disorders and lung diseases but also cardiac arrest [1,2,3,4].

PPG signal provides respiratory information, and it is a frequently utilized biomedical signal in RR estimation because the measurement method is convenient, low cost, and noninvasive [5,6]. PPG sensors measure changes in the blood volume from vessels near the skin, thereby acquiring information related to various vital signs [7]. The frequency range of a typical PPG signal is between 0.1 and 5 Hz. For accurate BP estimation, typical cut-off frequencies range from 0.3-4.5 Hz, whereas cut-off frequencies between 0.4–3 Hz are required for HR estimation [8,9]. In healthy adults, the RR ranges from 12–20 breaths per minute [10,11]. This enables the detection of most of the RR information at a relatively lower frequency than the frequencies associated with blood pressure and heart rate.

Previous studies related to PPG-based RR estimation have focused on two approaches: signal processing and deep learning-based RR estimation [12,13]. In signal processing-based approaches, frequency analysis is employed to extract from a PPG signal the components in the frequency domain that are associated with respiration. There are various frequency analysis methods such as the empirical mode decomposition (EMD) and wavelet transform [14,15,16,17]. In addition, methods such as respiratory-induced intensity variation (RIIV), respiratory-induced amplitude variation (RIAV), and respiratory-induced frequency variation (RIFV) [12,18] detect the optimal frequency band by decomposing the PPG signal, which modulates the PPG signal caused by respiration.

With the advancement of deep learning technology, RR estimation methods using various network structures have been proposed. For a robust estimation, Osathitporn et al. [19] proposed an end-to-end convolution neural network (CNN) with a residual block. They used three convolution blocks in parallel to extract various features related to respiration. All blocks in their network were composed of 1-D convolution blocks, leading to a reduction in the network size. Chowdhury et al. [20] also proposed a lightweight deep learning network for RR prediction. They added a projection layer at the front of the network to reduce the size of the input, followed by a residual module with depth-wise separable convolution blocks for a lightweight structure [21]. Spiking neural networks (SNNs) have recently attracted the attention of researchers, as an alternate to the lightweight deep neural networks (DNNs) for real-time monitoring, owing to their low computational cost and high energy efficiency [21,22,23,24].

**Figure 1:**
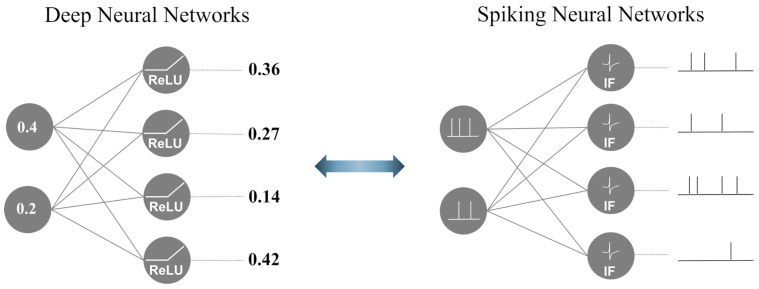
The functional difference between DNNs and SNNs.

SNNs are brain-inspired third-generation models that mimic neuronal dynamics [25]. Figure 1 illustrates the differences between a DNN and SNN. In contrast to DNNs, which propagate real-valued output, SNNs employ a discrete event-driven action potential called ‘spike trains’ as a temporal input and output. To generate spike trains as inputs for SNNs, real-value inputs are converted using various spike-encoding methods to transmit information [26,27,28]. Traditional spike-encoding schemes are classified into two categories: rate and temporal encoding. These encoding methods are widely used in SNN studies to convert visual information into spike trains. Rate encoding employs the probabilistic approach of the Poisson process, where a spike probabilistically occurs through a stimulus, such as pixel values in image data or the power spectrum in the frequency domain of time-series data. In contrast, temporal encoding focuses on the timing of the spike occurrence rather than the frequency of the encoding information. To manage the temporal spike trains, a biological spiking neuron model was incorporated into the SNNs. Temporal information is transmitted through accumulation and firing based on a threshold value in the spiking neuron, which leads to output spike trains that are directed towards the next neurons.

SNNs suffer from information loss during the encoding process [29,30,31]. In addition, the non-differential characteristics of spike trains impose limitations on learning in SNNs. Therefore, SNNs have been studied mainly in classification rather than regression fields [32]. Recently, multiple studies have been conducted to combine the learning mechanisms and network structures of SNNs and DNNs for achieving a performance comparable to that of DNNs while maintaining energy efficiency. Sengupta et al. [33] proposed a deep spiking neural network (DSNN) with VGG [34] and residual architectures [35]. To overcome the inherent challenges of SNNs, they adopted an artificial neural network for the spiking neural network conversion method. They conducted pretraining under the ReLU-based ANN structure, and then converted the weights for the initialization of the SNNs; this will preserve the weights from the ANN, minimize information loss, and enhance performance. Despite the loss of information, Guerrero et al. [36] evaluated an event-based regression problem using a DSNN. They utilized the temporal relationship of continuous spike patterns via a recurrent neural network (RNN)-like neuron model to demonstrate the possibility of regression fields.

In this study, we proposed an SNN framework for RR estimation. In addition to the energy efficiency advantages of SNNs, an SNN architecture combined with CNNs was adopted to ensure accurate performance. The contributions of this study are summarized as follows:

- We designed an end-to-end SNN architecture using a feedback-based neuronal model. To the best of our knowledge, this is the first regression study that applies end-to-end SNN to real-world PPG data.
- We employed a direct encoding method to convert real-valued PPG segments into spatial–temporal spike trains. We generated explainable spike trains for RR estimation via trainable convolution blocks with a biological neuron model.
- We compared the proposed model with other deep learning methods and demonstrated that the proposed model had an accuracy comparable to that of existing DNN models while being more energy efficient.

The remainder of this paper is structured as follows: Section 2 illustrates the proposed network structure and methodology. Section 3 presents the experimental settings for evaluating the proposed model and compares the benchmark results with other DNN architectures. Section 4 discusses further details of the proposed model and analyzes the experimental results. Finally, Section 5 concludes the paper and suggests directions for future research.

## 2. Materials and Method

### 2.1. Data and Preprocessing

To evaluate the proposed model, the BIDMC PPG, and Respiration dataset [37] from PhysioNet [38], containing signals extracted from the MIMIC II matched waveform database [39], was used. The dataset consisted of 53 recordings of PPG and impedance respiratory signals acquired from adult patients aged 19–90 years at the Beth Israel Deaconess Medical Center (Boston, MA, USA). Each recording was made for 8 min and sampled at 125 Hz. The dataset was collected by an analog-to-digital converter (ADC) with 16-bit precision.

In this study, reference RR was obtained from the annotations of the dataset, which were sampled at 1 Hz. To minimize the influence of other components, a bandpass filter with a cutoff frequency between 0.1–0.6 Hz (6–36 breaths per minute) was used in the extraction of respiratory information. Furthermore, to ensure sufficient data for training and validation, we applied a data augmentation strategy. We augmented the data by overlapping the PPG signals at 1 s intervals. For each 16 s PPG segment, we obtained the next PPG segment overlapped by shifting 125 data points. As a result, the number of segments was increased 15 times through the data augmentation.

### 2.2. Spiking Neuron Model

Various biologically plausible spiking neuron models, such as the Hodgkin–Huxley (HH), Izhikevich, integrate-and-fire (IF), and leaky integrate-and-fire (LIF), have been proposed to transmit information converted into spike trains [40,41,42]. In particular, the IF and LIF neuron models have been employed in numerous studies to leverage the advantages of both biological plausibility and computational efficiency. In this section, we describe the two neuron models used in the proposed network: soft-reset IF and recurrent IF neuron models.

**Figure 2:**
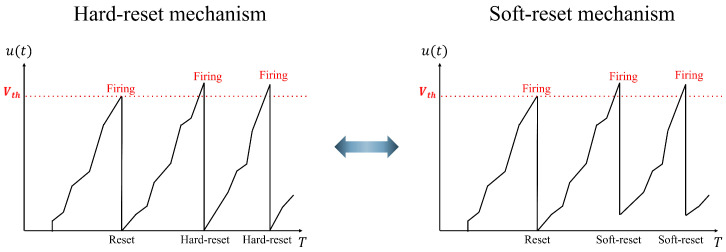
Difference in the operation of the hard-reset and soft-reset mechanisms in an IF neuron.

(1) Soft-reset IF neuron model: This neuron model is utilized in the spike encoder to minimize the information loss during spike conversion. Figure 2 shows the differences between the hard and soft-reset mechanisms. In the hard-reset approach, the membrane potential is reset to the reset voltage regardless of whether it exceeds the threshold. However, in the soft-reset approach, the membrane potential is initialized with a specific voltage that exceeds the threshold. The soft-reset IF neuron model is defined as follows:

(1) $$s_{i+1}\left(t\right)=\left\{\begin{array}{cc} 1, & \mathrm{if}\;u_{i+1}\left(t\right)-V_{th}\geq 0\\ 0, & \mathrm{if}\;u_{i+1}\left(t\right)-V_{th}<0 \end{array}\right.$$

(2) $$u_{i+1}\left(t\right)=u_{i+1}\left(t-1\right)\left(1-s_{i}\left(t\right)\right)+\left(u_{i+1}\left(t-1\right)-V_{th}\right)s_{i}\left(t\right),$$

(3) $$u_{i+1}\left(t+1\right)=u_{i+1}\left(t\right)+W_{i,i+1}s_{i}\left(t+1\right).$$

Equation (1) explains the Heaviside step function for spike activation. $s_{i+1}\left(t\right)$ denotes the output spike train, $u_{i+1}\left(t\right)$ is the membrane potential at the $t_{th}$ time step in the ${\left(i+1\right)}_{th}$ layer, and $V_{th}$ is a constant threshold voltage set as hyperparameter. If the membrane potential reaches the threshold, an output spike train is fired. Equation (2) expresses the soft-reset mechanism, where the membrane potential $u_{i+1}\left(t\right)$ depends on the Equation (1). If the spike is fired, the reset voltage is set to the difference between the previous membrane potential and the threshold; otherwise, it is maintained at the current value. Equation (3) expresses the IF neuron model, where $W_{i,i+1}$ denotes the trainable weights between the $i_{th}$ and ${\left(i+1\right)}_{th}$ layers.

(2) Recurrent IF neuron model: The conventional IF neuron model in Equation (3) accumulates the temporal information of the spike trains for sequential processing. However, there is no direct dependency among the time instances. In other words, updating the neuron from the current timestep is not influenced by the information from the previous timesteps. We utilized the feedback-based IF neuron model to incorporate information from the previous timestep and update of the current state of the neuron, whose mathematical model is defined in Equation (4):

(4) $$u_{i+1}\left(t+1\right)=u_{i+1}\left(t\right)+W_{i,i+1}s_{i}\left(t+1\right)+W_{rec}s_{i+1}\left(t\right),$$

where $W_{rec}$ denotes the recurrent weight of the (i+1)th layer. It leverages the relationship between the time instances by merging the information from the current spike trains in the (i+1)th layer with the previous spike trains in the *i*th layer.

### 2.3. Spike Encoding

Spike encoding is a crucial step in the processing of real-valued data using spike trains. The effective conversion of information into spike trains with minimal loss is crucial to the performance of the SNN model. Rate and temporal encoding methods have demonstrated good performance in classification tasks. Nevertheless, these traditional methods have limitations owing to information loss during the encoding process. In this study, we used a combination of direct encoding approaches and convolution to minimize the loss between the model prediction and its ground truth, enabling the direct conversion of PPG segments into spike trains without the need for additional processing steps.

We adopted a single-layer trainable 1D-CNN to encode information specifically related to respiration. The proposed method efficiently encoded only the respiratory information by extracting the temporal features of the PPG signal, which were obtained via trained convolution filters. Spike trains were generated through the accumulation and firing of respiratory information using a soft-reset IF neuron model that enables encoding with minimal loss.

### 2.4. Surrogate Gradient Learning

The most critical challenge in training SNNs is the non-differential problem of the spike activation function [43]. Backpropagation learning, which relies on differentiation, is typically used in the standard learning procedure for DNNs. However, the Heaviside step function was used for spike activation, as described in Equation (1). It imposes constraints on backpropagation learning owing to its non-differentiability at the instance when $u_{i+1}\left(t\right)$ equals $V_{th}$. To address this limitation, a surrogate gradient learning method is proposed, which introduces a differentiable surrogate function that approximates the behavior of the discontinuous Heaviside step function. The surrogate function enables the utilization of optimization techniques based on gradients, thereby facilitating the training process for SNNs, as described in Equation (5) with the first derivative.

(5) $$S\left(x\right)=\frac{x}{\alpha \sqrt{1+x^{2}}}+\frac{1}{2},$$

(6) $$S^{\prime }\left(x\right)=\frac{1}{\alpha {\left(1+x^{2}\right)}^{\frac{3}{2}}},$$

where *x* denotes the $u_{i+1}\left(t\right)-V_{th}$, and α is the scaling parameter to adjust the slope.

**Figure 3:**
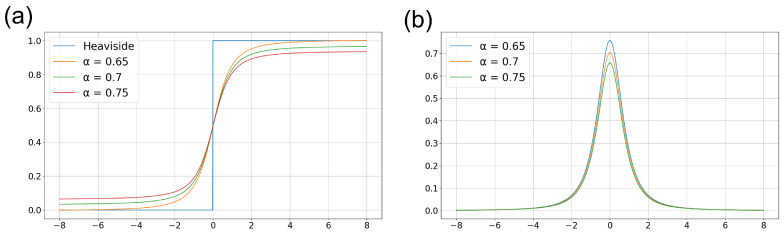
Surrogate functions for approximating the Heaviside step function. (**a**) Its original functions corresponding to the parameter α in Equation (5) and (**b**) its derivative function.

Figure 3a illustrates the Heaviside step function and approximate functions corresponding to various parameter values. The parameter α is experimentally chosen as 0.65, which best approximates the Heaviside step function. Figure 3b depicts the gradient functions corresponding to the functions shown in Figure 3a. By replacing the non-differentiable spike activation function with the proposed surrogate function, the network can be effectively trained in an approximated environment.

### 2.5. Network Structure

**Figure 4:**
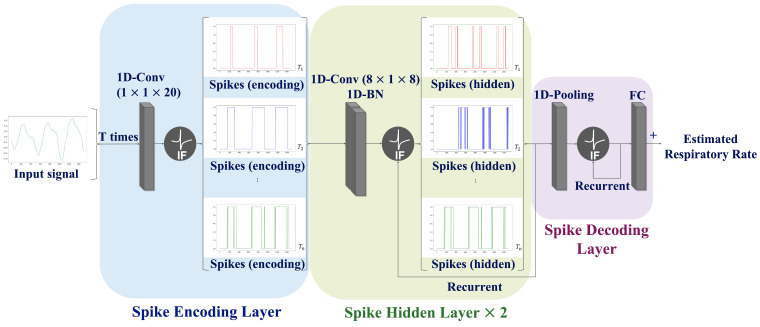
Schematic diagram of the proposed network structure. The proposed network comprises a spike encoding layer (blue box), spike hidden layer (green box), and spike decoding layer (purple box). Note that the spike hidden layer was employed iteratively twice.

To overcome the limitations of SNN prediction, a CNN-SNN architecture is proposed that combines convolution operations with the SNN architecture. The proposed network consists of three layers: a spike-encoding layer, spike-hidden layer, and spike-decoding layer; the spike hidden layer was employed iteratively twice. The information regarding the input signal is continuously processed across the *T* time steps within the network to ensure an accurate interpretation of the input signal. Consequently, the average of the output values over the *T* time steps is calculated to predict the RR. The proposed network paradigm is illustrated in Figure 4.

**Spike Encoding Layer:** In traditional encoding methods, the input data are transformed into spike trains before being fed into neural networks. In contrast, this study employs a trainable machine learning-based encoding method that directly utilizes a neural network for spike conversion. The features related to respiration were extracted through convolution operations. Subsequently, the soft-reset IF neurons described in Equations (1)–(3) was employed to generate spike trains.

**Spike Hidden Layer:** This layer includes two convolution blocks Each convolution block comprises a 1-D convolution, batch normalization, and recurrent IF neurons. The number of hidden layers was experimentally determined since the increase of the number of hidden layers causes a loss of accuracy.

**Spike Decoding Layer:** This layer is composed of a pooling layer, recurrent IF neurons, and a fully connected layer. A pooling operation was applied to aggregate the information and simultaneously reduce the number of spatial features. The respiratory information converted into spike form from the hidden layer was analyzed to estimate the RR.

### 2.6. Model Evaluation

To assess the model performance, we adopted Pearson’s Correlation Coefficient (PCC) and Mean Absolute Error (MAE) in breath per minute, depending on the sizes of the PPG segment [19]:

(7) $$PCC=\frac{\sum \left(Y-\bar{Y}\right)\left(Y^{\prime }-\bar{Y^{\prime }}\right)}{\sqrt{\sum {\left(Y-\bar{Y}\right)}^{2}\sqrt{\sum {\left(Y^{\prime }-\bar{Y^{\prime }}\right)}^{2}}}},$$

(8) $$MAE=\frac{1}{nW}\sum |Y-Y^{\prime }|,$$

where *Y* and $Y^{\prime }$ denote the true and estimated RR, respectively. $\bar{Y}$ and $\bar{Y^{\prime }}$ are the averages of the true and estimated RRs, respectively. W is the window size of the PPG segment.

Furthermore, we adopted the following methods to measure the energy efficiency of the proposed SNN and DNN model [44]:

(9) $$E\left(DNN\right)=\sum_{\ell =2}^{L}FL^{\ell }*E_{MAC},$$

(10) $$E\left(SNN\right)=FL^{\ell }*E_{MAC}+\sum_{\ell =2}^{L}FL^{\ell }*E_{AC},$$

where $FL^{\ell }$ denotes the number of floating-point operations at layer *ℓ*, $E_{MAC}$ is the energy consumption used for the multiply-–accumulate (multiplication and addition) operations, and $E_{AC}$ is the energy consumption for the accumulated (addition) operations. To count the number of floating point operations for each layer, we utilized the ptflops library. Furthermore, we assumed that the energy consumption for the addition process was 0.1 pJ and the multiplication process was 3.1 pJ, referring to [45].

We divided the training and test data at the subject level. From the BIDMC respiratory dataset, 40 subjects were randomly selected for the training process and 13 of them were used for the test process. Furthermore, a five-fold cross-validation method was applied during the training process. For the benchmark test, a CNN-LSTM model with three convolution layers and one LSTM layer [46], a CNN-RNN model with three convolution layers and one RNN layer [47], and a VGG-8 model were chosen [34]. The detailed parameter settings are presented in Table 1.

## 3. Experimental Results

The proposed model was implemented using the SpikingJelly framework based on the PyTorch library. The model training was conducted with a batch size of 16, a learning rate of 0.0005, and the Adam optimization method in an environment with an Intel Core i7-7700 CPU at 3.60 GHz and a GeForce RTX 4070ti GPU.

### 3.1. Model Accuracy

Table 2 lists the PCC and MAE performance corresponding to three different window sizes of the PPG segment. Experiments were conducted with 4, 8, and 16 timesteps of the spike trains. The proposed model demonstrated outstanding performance despite an increase in the number of time steps. The optimal performance was achieved when the time step T was set to 8. Generally, the mean firing rate plays a more critical role than the patterns of neuronal firing in SNN studies of classification problems [48,49,50]. Therefore, it was demonstrated that the performance improved with an increase in the timesteps during decision making. However, in the proposed model, longer timesteps of the spike trains did not improve performance.

Table 3 lists the PCC and MAE performances of the proposed model compared with those of other DNN approaches. The proposed model outperformed the CNN-RNN and VGG-8 models with MAE values of 1.37 ± 0.04 and 1.15 ± 0.07 bpm when the window sizes were 16 and 64, respectively. It also yielded better performance than the VGG-8 model, with an MAE of 1.23 ± 0.03 bpm at a window size of 32. The model of Osathitporn et al. [19] exhibited the best performance for the 16 and 32 window sizes with MAE of 1.34 ± 0.01 and 1.11 ± 0.01 bpm. The CNN-LSTM showed the best performance with MAE of 1.11 ± 0.03 bpm in 64 window size. However, the overall results of the proposed model achieved comparable performance with the CNN-LSTM, CNN-RNN and Osathitporn et al. [19] models.

**Figure 5:**
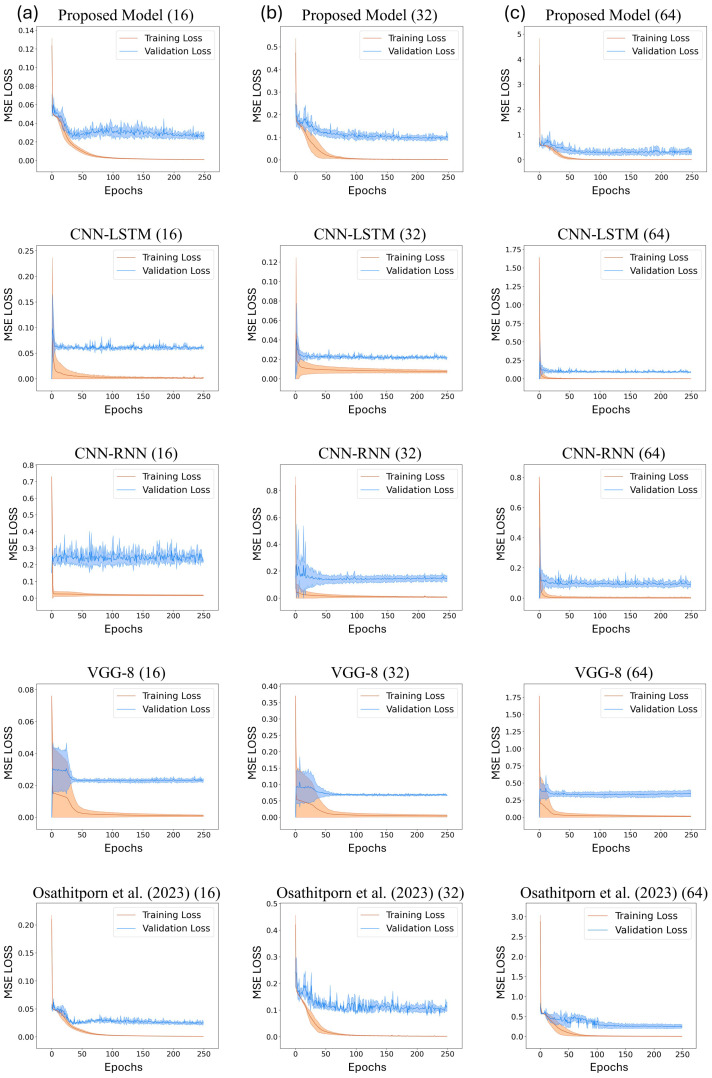
Train and validation losses about CNN-LSTM, CNN-RNN, VGG-8, and Osathitporn et al. [19] when the window size is (**a**) 16, (**b**) 32, and (**c**) 64. The standard deviations are represented with shades.

Figure 5a–c display the training and validation loss curves of the proposed model and the other DNN models with window sizes of 16, 32, and 64, respectively. The x-axis represents the training and validation epochs, and the y-axis represents the mean squared error (MSE) losses. The validation losses converge in all cases, and these curves validate the reliability of the results presented in Table 2. In particular, Figure 5c displays the optimal convergence compared to Figure 5a,b.

**Figure 6:**
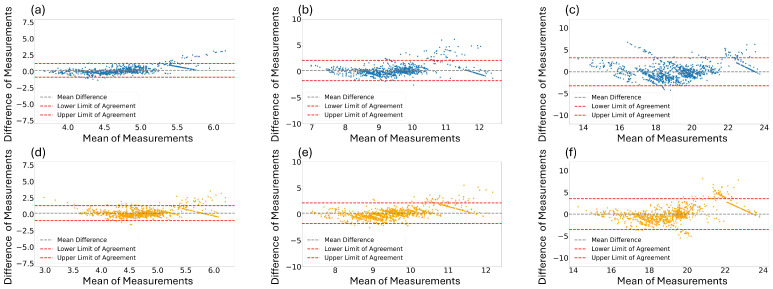
Bland–Altman graphs for the best performing models (**a**,**b**): Osathiporn et al. [19], (**c**): CNN-LSTM) and the proposed model (**d**–**f**) with window sizes of 16, 32, and 64. Note that the interval between the lower limit of agreement and the upper limit of agreement represents a 95% confidence interval.

In addition to the PCC in Table 4, we performed visualization to evaluate the reliability of each estimated RR. Figure 6 shows the Bland–Altman graphs between the estimated RR and ground-truth RR. Figure 6a,b illustrate the results for window sizes of 16 and 32 s using the best performing model, the Osathitporn et al. [19] network as indicated in Table 4. Figure 6c shows the results for a 64-second window size, using the CNN-LSTM network. Figure 6d–f visualize the results of the proposed model. The x-axis represents the average of two measurements, and the y-axis represents difference between the two measurements. For both the best performing models and the proposed model, the majority of the data points contain within the 95% confidence interval, demonstrating the reliability of the models.

### 3.2. Computational Cost and Energy Consumption

Table 4 presents the floating point operations per seconds (FLOPs) and energy costs for the proposed SNN model and other DNN models. DNNs utilize MAC operations as metrics for FLOPs, whereas the proposed model employs synaptic operations [51]. The proposed model showed comparable performance to other DNN models in terms of MAE performance. Furthermore, compared to the best performing model, it demonstrated 18.6, 18.7, and 64.6 times higher energy efficiency, and 1.05, 1.1, and 3.97 times lower floating-point operation counts for the window sizes of 16, 32, and 64, respectively.

## 4. Discussion

Our approach utilized a suitable network architecture to perform a regression test from time-series medical data, whereas most other SNN studies have focused on classification problems owing to their poor accuracy performance caused by information loss. In particular, a spiking neuron model was designed with a recurrent structure similar to RNNs [47], reflecting the spike information from previous time instances in the next one. Therefore, the temporal dependencies among the different time instances can be enhanced using recurrent spiking neurons with the feature extraction capabilities of CNNs. However, there is a limitation in learning long-term dependencies. To address this limitation, surrogate gradient learning has been proposed, which introduces a differentiable surrogate function that approximates the behavior of a discontinuous Heaviside step function. The surrogate function enables the utilization of optimization methods based on a gradient process, thereby conducting a training process for the SNNs.

**Figure 7:**
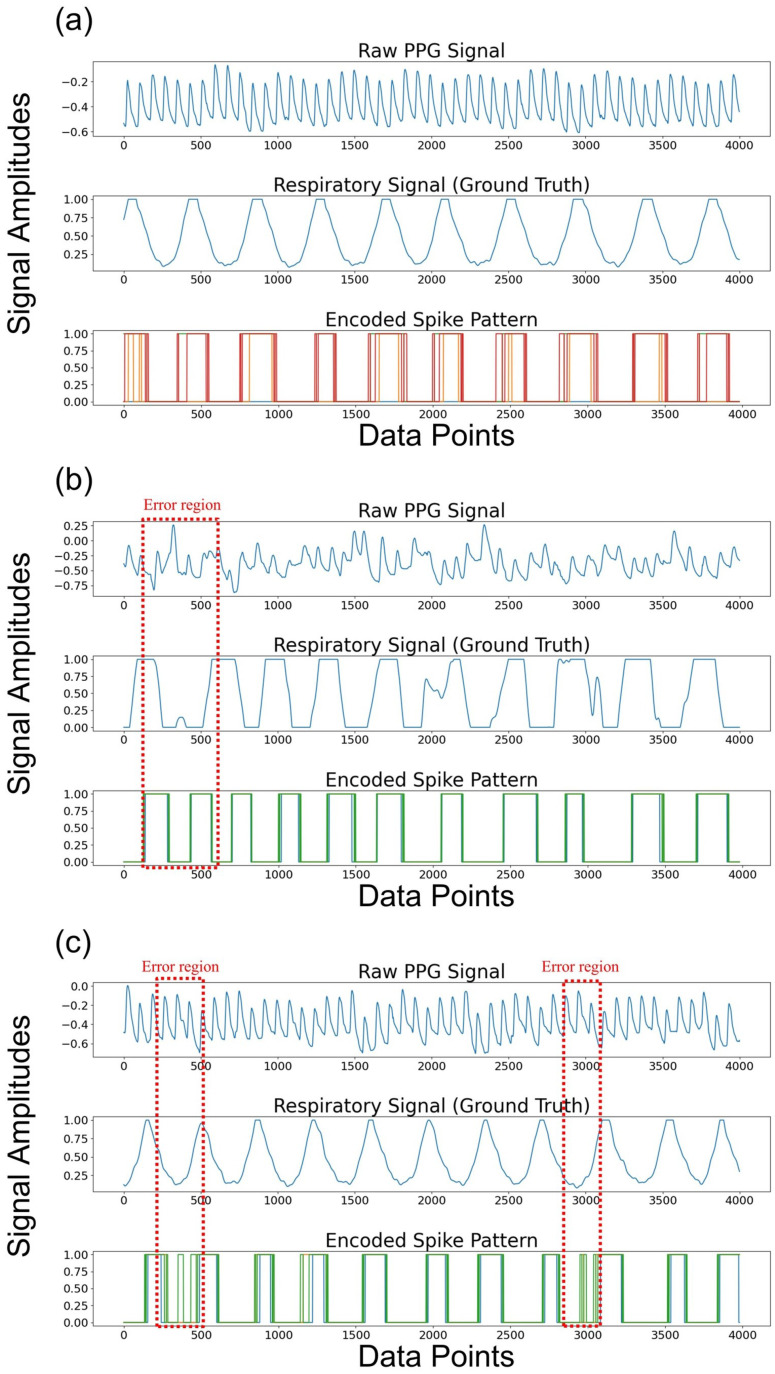
The raw PPG signals, true respiratory signals, and its encoded spike patterns from the PPG signal within 32-s window are visualized across (**a**) a clean PPG signal and (**b**), (**c**) noisy PPG signals. Note that the dotted red boxes depict the error regions.

To analyze the outstanding performance of the proposed SNN model, we visualized the spike patterns derived from the PPG signals (see Figure 7a–c). Figure 7 shows the results from randomly selected clean and noisy PPG signals. Figure 7a shows the raw PPG signals, its true respiratory signal, and the result of its spike encoding. The PPG signal was bandpass filtered into the respiratory band corresponding to 0.1–0.6 Hz. The respiratory information was perfectly captured in the spike pattern, which was generated around the peaks of the respiratory signal, indicating that it contained sufficient respiratory information. Furthermore, Figure 7b,c illustrate that the respiratory information was effectively represented, even with noisy PPG signals, except for the minor regions depicted in the red dashed box.

We set the number of time steps to eight. In other words, the CNN extracts the features from the PPG signals, and the process of generating spikes with the soft-reset IF neurons is repeated eight times. In this process, the membrane voltage value from the previous time step is carried over to the next time step, since the neuron’s membrane voltage is not reset at each time step. Consequently, spikes are produced at the valleys of the respiratory signal in noisy PPG signals despite the application of the bandpass filter to extract the respiratory information from the low-frequency components of the PPG signal, leading to the error. To minimize these errors, noise reduction methods such as smoothing filters could be applied. Furthermore, utilizing the multiple convolution layers can be also used to extract accurate respiratory information.

## 5. Conclusions

In this study, an SNN-based model for respiratory rate prediction was proposed and compared with deep learning models in terms of accuracy and energy cost. By enhancing the time dependency through the recurrent structure, the proposed model showed accuracy performance comparable to that of deep learning models such as CNN-LSTM, CNN-RNN, VGG-8, and state-of-the-art RR estimation networks, operating at a relatively low computational cost. As a result, the proposed model demonstrated its advantage in low power consumption with a maximum of 64.5, 52.7, 50.5, and 20 times lower energy cost compared to the deep learning models. Furthermore, the analysis of the spike patterns from the trainable spike encoder revealed that spikes were periodically generated corresponding to the respiratory patterns of the reference signals, which enhanced the reliability of the model. To the best of our knowledge, this is the first study to apply an end-to-end SNN architecture to the regression analysis of real-world PPG data, thereby validating its effectiveness in RR estimation.

## Figures and Tables

**Table 1:** Components of the proposed and benchmark models.

Model Components	Proposed Model	CNN-LSTM	CNN-RNN	VGG-8	Osathitporn et al. [19]
CNN Layers	3	3	3	5	5
LSTM Layers	-	1	-	-	-
RNN Layers	-	-	1	-	-
Dense Layers	2	1	1	3	3
CNN Filter Size	20/8/8	10/5/5	10/5/5	3/3/3/3/3	16/32/64/3/3
Activation Functions	IF/RLIF	Leaky-ReLU	Leaky-ReLU	Leaky-ReLU	Leaky-ReLU

**Table 2:** PCC and MAE performances corresponding to the different window sizes of PPG segment and time steps.

Time Steps (T)	Window Size (s) = 16		Window Size (s) = 32		Window Size (s) = 64	
	PCC	MAE (bpm)	PCC	MAE (bpm)	PCC	MAE (bpm)
T = 4	0.4980 ± 0.0287	1.5247 ± 0.0332	0.6074 ± 0.0064	1.3642 ± 0.0129	0.6219 ± 0.0111	1.3153 ± 0.0134
T = 8	**0.5695 ± 0.0319**	**1.3710 ± 0.0481**	**0.6360 ± 0.0070**	**1.2234 ± 0.0372**	0.6615 ± 0.0473	**1.1518 ± 0.0697**
T = 16	0.5383 ± 0.0343	1.4671 ± 0.0157	0.5447 ± 0.0117	1.3646 ± 0.0352	**0.6630 ± 0.0199**	1.2204 ± 0.0493

**Table 3:** Testing results of the proposed model compared with the other DNN models.

Model	Window Size (s) = 16		Window Size (s) = 32		Window Size (s) = 64	
	PCC	MAE (bpm)	PCC	MAE (bpm)	PCC	MAE (bpm)
CNN-LSTM	0.5926 ± 0.0355	1.3681 ± 0.0685	0.6864 ± 0.0342	1.1169 ± 0.0705	0.7077 ± 0.0245	**1.1116 ± 0.0343**
CNN-RNN	0.5233 ± 0.0113	1.4757 ± 0.0348	**0.6980 ± 0.0270**	1.1605 ± 0.0675	**0.7489 ± 0.0298**	1.1537 ± 0.0448
VGG-8	0.4577 ± 0.0329	1.4721 ± 0.1795	0.5305 ± 0.0312	1.4434 ± 0.0705	0.5007 ± 0.1199	1.4053 ± 0.0964
Osathitporn et al. [19]	**0.5945 ± 0.0142**	**1.3460 ± 0.0128**	0.6705 ± 0.0045	**1.1121 ± 0.0108**	0.6643 ± 0.0103	1.1321 ± 0.0150
Proposed model	0.5695 ± 0.0319	1.3710 ± 0.0481	0.6360 ± 0.0070	1.2240 ± 0.0372	0.6615 ± 0.0473	1.1518 ± 0.0697

**Table 4:** FLOPs and energy cost results of the proposed model compared with the other DNN models.

Model	Window Size (s) = 16		Window Size (s) = 32		Window Size (s) = 64	
	FLOPs (M)	Energy Cost (μJ)	FLOPs (M)	Energy Cost (μJ)	FLOPs (M)	Energy Cost (μJ)
CNN-LSTM	16.44	0.5260	44.38	1.4200	84.75	2.7120
CNN-RNN	31.99	1.0230	35.04	1.1210	69.26	2.2160
VGG-8	16.01	0.5523	28.19	1.0760	55.99	2.1250
Osathitporn et al. [19]	6.98	0.2233	13.44	0.4301	26.34	0.8428
Proposed model	**6.59 **	**0.0120**	**12.21**	**0.0230**	**21.32**	**0.0420**

## Data Availability

The data presented in this study are openly available in PhysioNet at https://doi.org/10.13026/C2208R.
